# Full-length antithrombin frameshift variant with aberrant C-terminus causes endoplasmic reticulum retention with a dominant-negative effect

**DOI:** 10.1172/jci.insight.161430

**Published:** 2022-10-10

**Authors:** Carlos Bravo-Pérez, Mara Toderici, Joseph E. Chambers, José A. Martínez-Menárguez, Pedro Garrido-Rodriguez, Horacio Pérez-Sanchez, Belén de la Morena-Barrio, José Padilla, Antonia Miñano, Rosa Cifuentes-Riquelme, Vicente Vicente, Maria L. Lozano, Stefan J. Marciniak, Maria Eugenia de la Morena-Barrio, Javier Corral

**Affiliations:** 1 Servicio de Hematología y Oncología Médica, Hospital Universitario Morales Meseguer, Centro Regional de Hemodonación, University of Murcia, Biomedical Research Institute of Murcia, CB15/00055-CIBERER, Murcia, Spain.; 2 Cambridge Institute for Medical Research, University of Cambridge, Cambridge, United Kingdom.; 3 Department of Cell Biology and Histology, Medical School, Biomedical Research Institute of Murcia, University of Murcia, Campus Mare Nostrum, Murcia, Spain.; 4 Structural Bioinformatics and High Performance Computing Research Group, Universidad Católica de Murcia, Murcia, Spain.

**Keywords:** Genetics, Hematology, Genetic diseases, Serpins, Thrombosis

## Abstract

Antithrombin, a major endogenous anticoagulant, is a serine protease inhibitor (serpin). We characterized the biological and clinical impact of variants involving C-terminal antithrombin. We performed comprehensive molecular, cellular, and clinical characterization of patients with C-terminal antithrombin variants from a cohort of 444 unrelated individuals with confirmed antithrombin deficiency. We identified 17 patients carrying 12 C-terminal variants, 5 of whom had the p.Arg445Serfs*17 deletion. Five missense variants caused qualitative deficiency, and 7, including 4 insertion-deletion variants, induced severe quantitative deficiency, particularly p.Arg445Serfs*17 (antithrombin <40%). This +1 frameshift variant had a molecular size similar to that of WT antithrombin but possessed a different C-terminus. Morphologic and cotransfection experiments showed that recombinant p.Arg445Serfs*17 was retained at the endoplasmic reticulum and had a dominant-negative effect on WT antithrombin. Characterization of different 1+ frameshift, aberrant C-terminal variants revealed that protein secretion was determined by frameshift site. The introduction of Pro441 in the aberrant C-terminus, shared by 5 efficiently secreted variants, partially rescued p.Arg445Serfs*17 secretion. C-terminal antithrombin mutants have notable heterogeneity, related to variant type and localization. Aberrant C-terminal variants caused by 1+ frameshift, with similar size as WT antithrombin, may be secreted or not, depending on frameshift site. The severe clinical phenotypes of these genetic changes are consistent with their dominant-negative effects.

## Graphical abstract

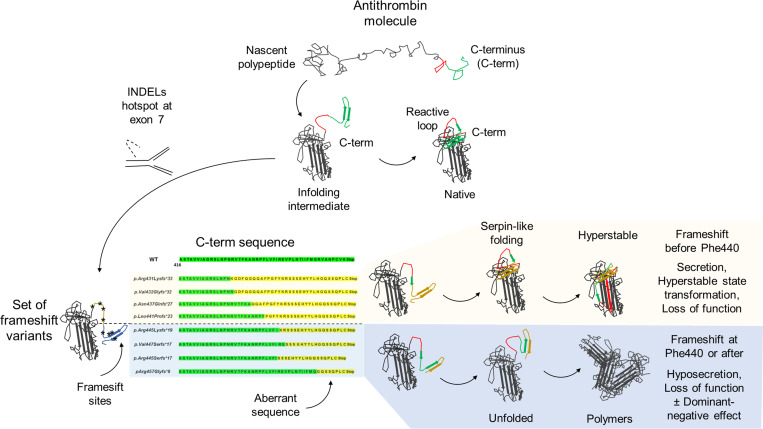

## Introduction

Antithrombin (AT) is a plasma glycoprotein of hepatic origin and a key endogenous anticoagulant that inhibits thrombin and many other coagulation serine proteases (1–3). AT deficiency (defined as AT activity <80% normal) is associated with an increased risk of venous thromboembolism (VTE). Congenital AT deficiency, the first major thrombophilia to be reported and the most severe to date (3, 4), is a rare, dominant inherited disorder (0.02–0.2%) (5). Patients with AT deficiency have severe (early and recurrent) VTE, which can be fatal or require long-term anticoagulant treatment (3, 4).

AT is encoded by the *SERPINC1* gene (1q23-q25.1; 13.5 kb long; *n* = 7 exons and 6 introns) (6). Newly synthesized AT polypeptides have 464 aa and require the N-terminal signal (*n* = 32 aa) to be cleaved. Mature AT (*n* = 432 aa; 58 kDa) (2) has 4 asparagine (Asn) residues susceptible to N-glycosylation (Asn128, Asn167, Asn187, and Asn224), and 6 cysteine (Cys) residues that form 3 disulfide bonds (Cys40-160, Cys53-127, and Cys279-462) (7).

From a structural perspective, AT is a serpin (i.e. a serine-protease inhibitor) (7). As with other members of this protein superfamily, such as alpha-1-antitrypsin (AAT), it possesses 3 β sheets (A–C), 9 α helices (A–I), and a reactive center loop (RCL), enabling efficient protease inhibition by a mousetrap-like mechanism (8). Unlike most serpins, the RCL of native AT is only partially exposed, and it only becomes completely accessible once heparinoids bind to the heparin-binding site (HBS), increasing inhibitory activity up to 1000-fold (9).

More than 400 pathogenic variants in *SERPINC1* have been described to date (10). Molecular defects show notable heterogeneity (4, 11, 12). Classically, AT deficiency has been divided into type I (quantitative) and type II (qualitative) defects. Type I deficiency is commonly due to heterozygous insertion-deletion variants (INDELs), as well as nonsense, splicing, or structural variants, which normally lead to severe loss of AT in plasma and early or recurrent thrombosis (12). Type II deficiency is mainly due to missense gene variants, with a milder impact on protein structure and secretion, but with impaired function that causes heterogenous clinical consequences (13).

The analysis of natural mutants of AT has helped identify functional and structural domains of this and other serpins (8, 14, 15). In contrast to RCL and HBS, the distal part of the C-terminus of AT (p.Val432-Lys464) has no known functionality. However, evidence from studies of other serpins (16–18) and from case reports of *SERPINC1* mutations involving AT C-termini (10) support that it might be necessary for the folding and secretion of the molecule. Noteworthy, the C-terminus remains dissociated from the serpin backbone in a relatively unstructured state until the last folding steps. Indeed, after insertion of strand 5A for completion of β-sheet A, 1 of the last events is the incorporation of the C-terminus, which results in both completion of β-sheet C (by the insertion of strand 1C [s1C]) and, subsequently, β-sheet B (after incorporation of the strand 4B–strand 5B [s4B–s5B] hairpin) (19).

Based on these observations, the insertion of the C-terminus of AT might play a crucial role not only in the physiologic state but also in morbid conditions caused by gene variants (19). Remarkably, there is considerable biological and clinical heterogenicity among C-terminal AT variants (10–12). Most of these are missense changes causing type II deficiencies not directly involving the RCL, HBS or so-called pleiotropic effect (II PE) mutations (13). In those cases, either inhibitory activity or heparin binding of AT could be indirectly impaired because they facilitate the transition to a latent conformation (20), but it seems that defective protein synthesis, folding, degradation, and/ or secretion could also be involved (13). Nevertheless, several INDELs leading to frameshift at the AT C-terminus have been described (21–27). As opposed to the majority of type II PE mutants, most INDELs generate type I deficiency, some of them with a dramatic reduction in AT secretion and activity (≤40%), higher than that expected for a heterozygous mutation in *SERPINC1* (40–60%). These observations led us to hypothesize that some variants might impair secretion of WT AT in a dominant-negative manner (28), as has recently been shown for other serpins, such as AAT and C1-inhibitor (29, 30).

In the present study, we aimed to characterize the molecular and clinical consequences of different variants affecting the C-terminus of AT. Our work confirms that biological and clinical heterogeneity of C-terminal AT variants correlates with their localization and suggests a key structural relevance of this region in AT and, potentially, in all serpins. Our study also shows for the first time, to our knowledge, that a set of frameshift, C-terminal AT variants causes severe type I deficiency by a dominant-negative mechanism, consisting of protein polymerization and endoplasmic reticulum (ER) retention. The correlation between molecular and clinical phenotypes could be of prognostic value, and these findings open the possibility of evaluating the impact of targeted therapies.

## Results

### Molecular and clinical characterization of patients with SERPINC1 variants involving the C-terminus of AT.

We identified 17 patients (3.8% from our cohort of 444 patients with AT deficiency) who carried 12 different *SERPINC1* variants affecting the distal region of the C-terminus of AT, all in exon 7 and not affecting the RCL (p.Val432-Lys464) (Table 1 and Supplemental Figure 1; supplemental material available online with this article; https://doi.org/10.1172/jci.insight.161430DS1). Eight variants were missense, and the other 4 were small INDELs. Two mutations were recurrent: p.Arg445Serfs*17 (*n =* 5) and p.Pro439Thr (*n =* 2). Five variants had not been previously reported, to our knowledge: p.Thr433Ser, p.Pro439Ala, p.Phe440Serfs*4, p.Pro461Ser, and p.Val458_Cys462delinsGly.

Biological and clinical characterization of these cases revealed phenotypic dimorphism, depending on the type of variant and its localization within the molecule (Table 1). Five missense variants (p.Phe434Leu, p.Arg438Gly, p.Pro439Thr, p.Pro439Ala, and p.Pro461Ser), mainly located at s1C, caused type II PE deficiency, with increase of latent AT in plasma and a moderate to severe thrombotic phenotype (Supplemental Figure 2). One of these cases, presenting the p.Pro439Thr variant, alternated normal and defective anticoagulant activities in a transient manner, with fluctuating levels of the latent form (31). On the other hand, 7 gene variants, including all INDELs (p.Thr433Ser, p.Phe440Serfs*4, p.Leu441Pro, p.Ile444Metfs*19, p.Arg445Serfs*17, p.Gly456Arg, p.Val458_Cys462delinsGly), mainly located at s4B–s5B, caused type I deficiency, and defined severe, early VTE (Table 1). One missense mutation causing quantitative deficiency, p.Gly456Arg, was associated with the identification in plasma of disulfide-linked, high–molecular weight complexes in both denaturing nonreducing and native conditions (32).

### Small deletion causing 1+ frameshift, p.Arg445Serfs*17, generates a full-length AT variant with an aberrant C-terminus leading to severe type I deficiency.

**Figure 1:**
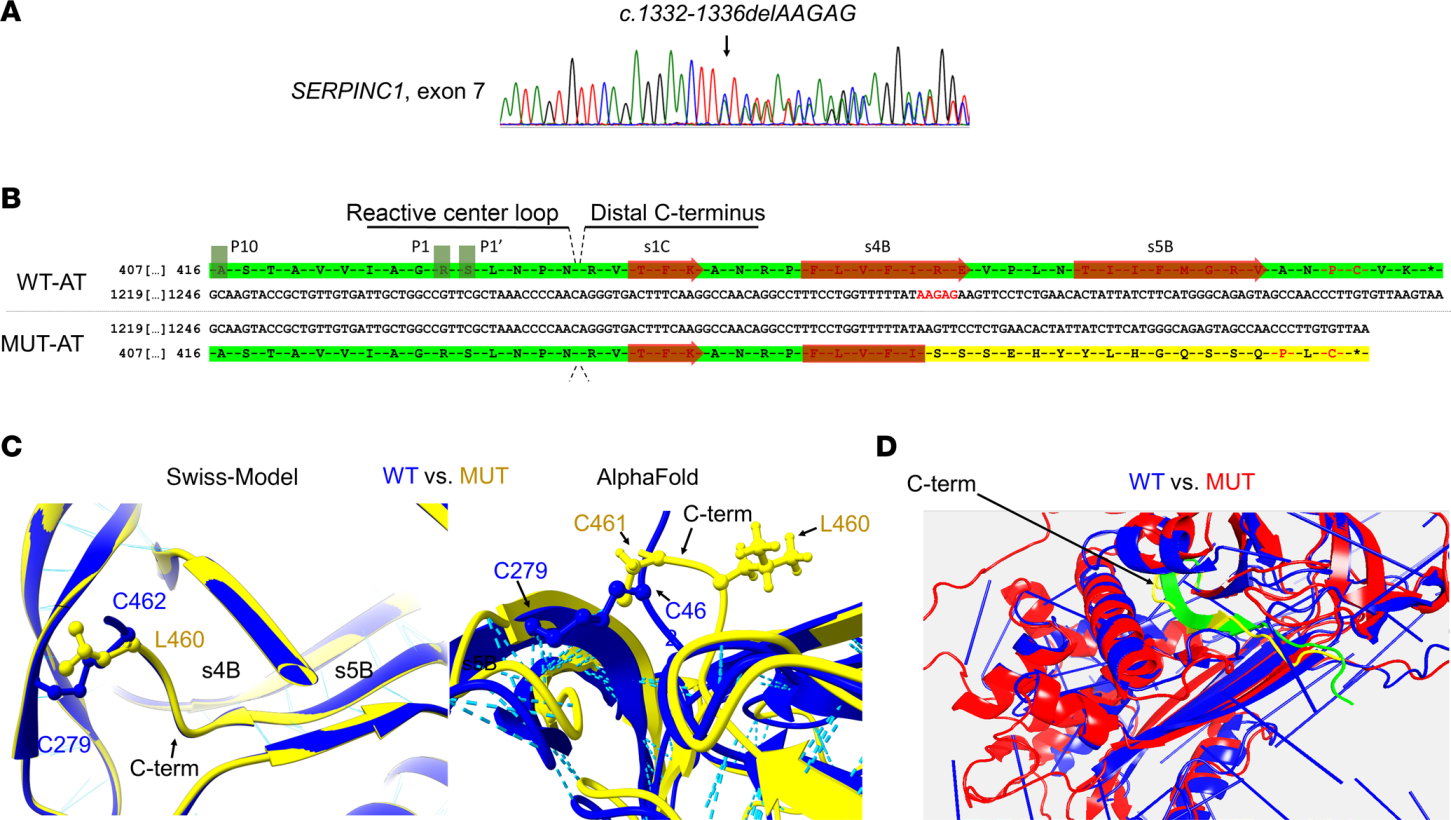
Comparative analysis of in silico predictions and molecular dynamics simulation for p.Arg445Serfs*17 variant versus WT AT. (**A**) Chromatograph (reverse sequence) detecting the gene variant in exon 7 of *SERPINC1*. The small deletion c.1332-1336delAAGAG is highlighted (arrow). (**B**) Nucleotide and primary sequence of p.Arg445Serfs*17 and WT AT. The nucleotide deletion and the aberrant C-terminal sequence are highlighted in red and yellow, respectively. Conserved Pro and Cys C-terminal residues are highlighted in red. (**C** and **D**) Graphic comparison of structures of p.Arg445Serfs*17 and WT AT through Swiss-Model and AlphaFold, visualized with UCSF Chimera (**C**), and molecular dynamics simulation (**D**). Note the similarities in C-terminal s4B-s5B segments, hydrogen-bond and disulfide-bond formation. Molecular dynamics simulation maximum time setup was 680 ns. C-term, C-terminus; MUT-AT, mutant antithrombin.

Among the previously C-terminal AT variants identified in our cohort, we focused on c.1332-1336delAAGAG p.Arg445Serfs*17, a small deletion of 5 nucleotides in exon 7 of *SERPINC1* causing a 1+ frameshift affecting 17 aa. Described by Millar et al in 1993 (21) in a Spanish thrombophilic family, it was the most frequent C-terminal variant identified in our cohort (*n =* 5) and 1 of the most severe (Table 1 and Figure 1A). All of our case patients also were Spanish and came from the same geographic region. Next-generation sequencing of the whole *SERPINC1* gene performed in 3 of 5 cases revealed a common haplotype in *SERPINC1*, suggesting a founder effect (Supplemental Table 1).

Prediction of the resulting aberrant C-terminus caused by this frameshift revealed an abnormal aa sequence, however, that did not generate a premature stop codon, so the mutant allele might encode a near full-length protein (Figure 1, A and B). Moreover, 2 key residues of the WT C-terminus, Pro459 and Cys461, presumed to be crucial for AT folding, secretion, and function (18, 20), were preserved in the aberrant C-terminus (Pro461 and Cys462) (Figure 1B). All the different in silico modeling algorithms tested, including the machine-learning system AlphaFold and molecular dynamics simulation, suggested that the aberrant-C-terminal variant might form similar secondary and tertiary structures, enabling the formation of the C-terminal disulfide bond between Cys461 (from the aberrant C-terminal end) and Cys279 (from the WT skeleton) (Figure 1, C and D).

Nevertheless, p.Arg445Serfs*17 was not secreted into plasma and so caused severe type I AT deficiency. In fact, most of the carriers of the defective allele from our cohort constitutively presented with drastic reductions of AT in plasma, with anti–activated factor Xa (anti-FXa) activity of 40% or less (median anti-FXa: 41.5%; IQR: 36.5–50%; minimum: 30%, maximum: 50%) (Table 1), which is unexpected for a classical type I mutation in the heterozygous state, including typical nonsense mutants.

### Recombinant p.Arg445Serfs*17 has reduced secretion and provokes dilation and fragmentation of the ER.

**Figure 2:**
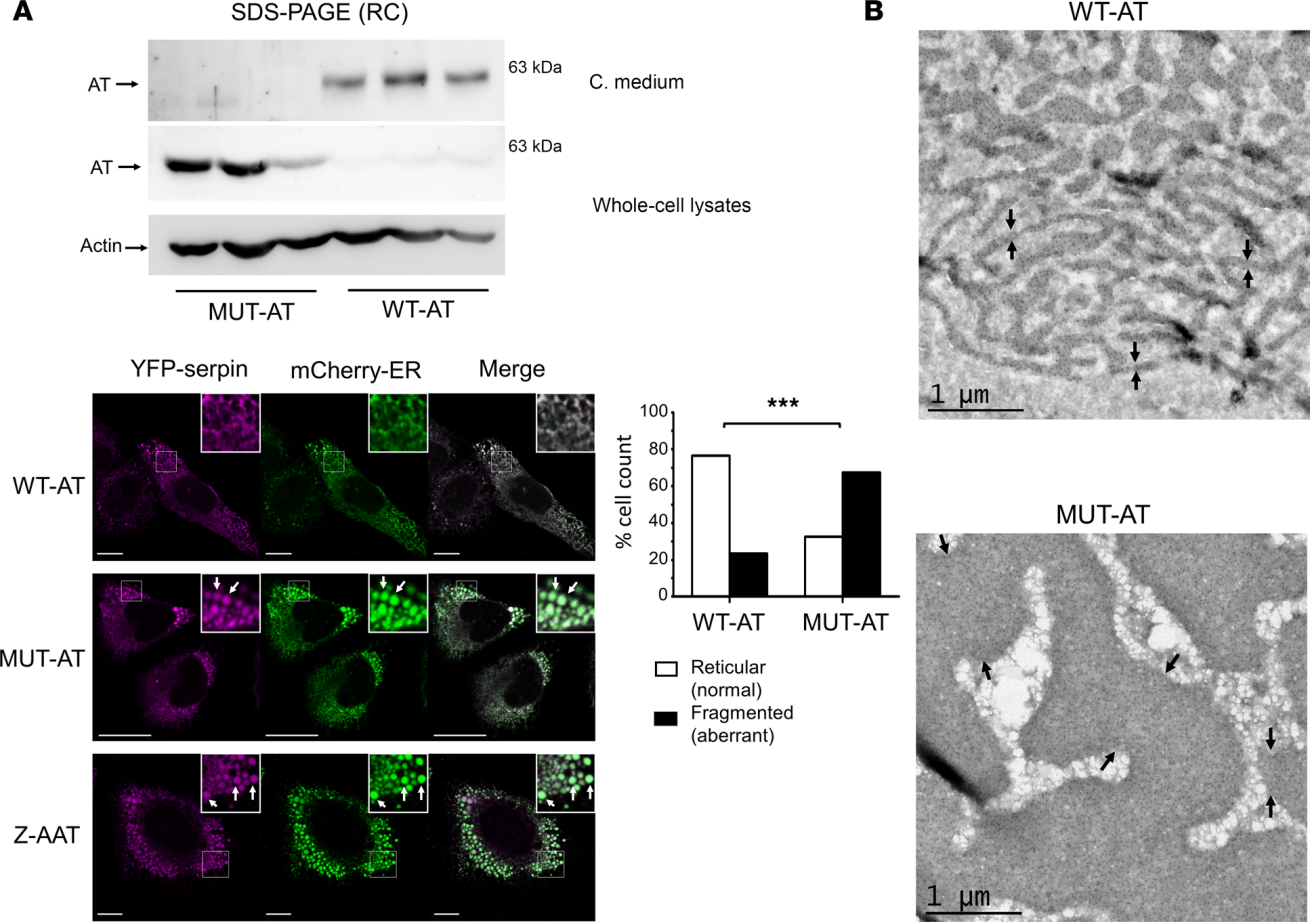
Morphological analysis of the intracellular consequences of recombinant p.Arg445Serfs*17 variant. (**A**) Western blot for AT in reducing conditions, SDS-PAGE of conditioned medium (C. medium) and whole-cell lysates of CHO cells transfected with untagged WT and mutant (MUT) AT plasmids, and confocal micrographs of CHO cells transfected with YFP-tagged WT or mutant AT, or Z-AAT, and with an ER marker plasmid (mCherry-ER). White-bordered boxes denote regions magnified and inset in the top-right of panels. Scale bars: 10 μm. Note the vesicular ER morphology (white arrows) in MUT-AT and Z-AAT, but not in WT-AT, cells. Quantitative analysis revealed that the proportion of dilated or fragmented-ER cells was significantly higher in MUT-AT than WT-AT cells (67.5% vs. 23.5%; Pearson’s χ^2^, *P <* 0.001). Note that the intensity acquisition parameters are not comparable in these experiments. (**B**) Transmission electron micrographs (AT gold immunostaining) of HEK-EBNA cells transfected with untagged WT and mutant (MUT) AT plasmids. Scale bars: 1 μm. Note the massive cisternae dilation of the ER (delimited between black arrows), due to mutant protein accumulation. ***P < 0.001. RC, reducing conditions.

To clarify the molecular mechanism underlying p.Arg445Serfs*17, we used a eukaryotic system of ectopic expression. We generated the p.Arg445Serfs*17 variant by site-directed mutagenesis and transiently expressed the recombinant plasmid in human embryonic kidney expressing the Epstein Barr nuclear antigen 1 (HEK-EBNA) and Chinese hamster ovary (CHO) cells. WT-AT plasmid was used as control. Forty-eight hours after transfection, we confirmed by Western blot that, although the electrophoretic mobility of WT and mutant AT were similar, the secretion of the C-terminal variant AT, compared with the WT, was severely reduced due to intracellular retention (Figure 2A).

To further characterize the pathogenic mechanism associated with the impaired secretion of p.Arg445Serfs*17, expression constructs encoding yellow fluorescent protein (YFP)–tagged mutant and WT AT were generated. As previously described for AAT, findings from of the resulting YFP-serpin plasmids can be extrapolated to the untagged serpins. The ER marker mCherry-ER plasmid and YFP-tagged mutant or WT AT constructs were cotransfected into the CHO line. The YFP–Z form of AAT (Z-ATT) was also cotransfected with mCherry-ER as a model of serpin polymerization and retention within the ER (33). After 48 hours of transfection, operator-blind counting of reticular ER (normal) and dilated or fragmented ER (aberrant) cells was performed. YFP-mutant AT caused a significant dilation and fragmentation of the ER, unlike YFP-WT AT. Quantitative analysis revealed that the proportion of dilated or fragmented-ER cells was significantly higher in cells transfected with the mutant plasmid than in those transfected with the WT plasmid (67.5% vs. 23.5%; *P <* 0.001). These morphologic features were similar to that of YFP-Z-AAT (Figure 2A).

Additionally, we performed ultrastructural analysis by transmission electron microscopy and gold immunostaining of AT on transiently transfected HEK-EBNA. After 96 hours of transfection, the ER of cells transfected with variant AT had severe morphologic changes (namely, massive cisternae dilation) and a high electron density due to AT accumulation, findings that were not observed in cells transfected with WT AT (Figure 2B).

### Recombinant p.Arg445Serfs*17 has a dominant-negative effect on WT AT.

**Figure 3:**
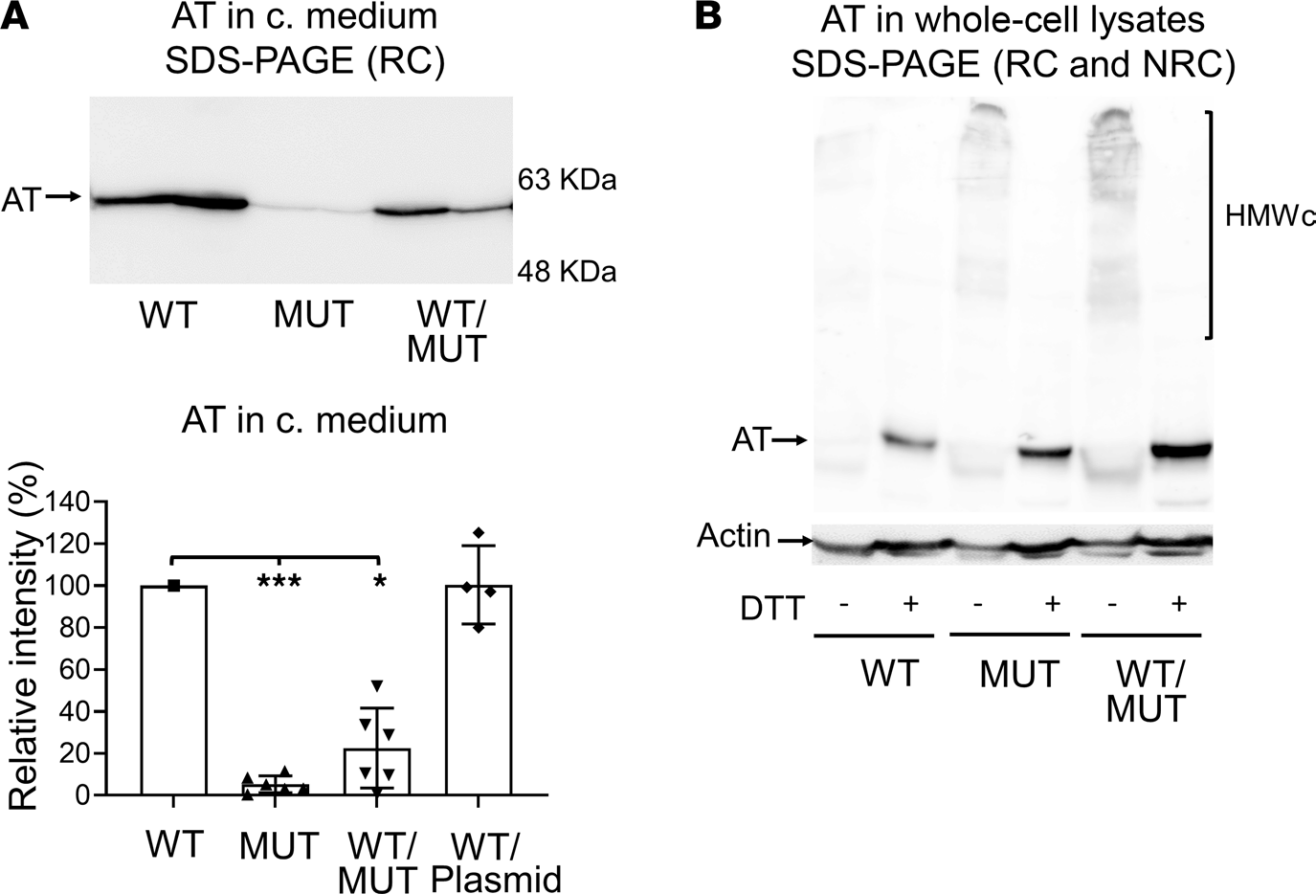
Characterization of p.Arg445Serfs*17 dominant-negative effect on WT AT in a recombinant model of transient expression in HEK-EBNA cells. (**A**) Western blot for AT in conditioned medium (c. medium) in reducing conditions (RCs), SDS-PAGE, and densitometric analysis of relative (vs. WT) protein secretion, estimated with ImageJ software. Variant (MUT) secretion was severely reduced, compared with the WT protein (mean relative secretion, 5.3%; SD 4.0%). Cotransfection experiments (WT/MUT) showed that the mutant plasmid had a dominant-negative effect on WT AT (mean relative secretion, 22.5%; SD 19.1%). WT/Plasmid denotes cotransfection of WT plasmid and control plasmid not containing AT cDNA insert (*n* = 7 experiments). *P < 0.05, ***P < 0.001 by Mann-Whitney *U* test. (**B**) Western blot for AT of whole-cell lysates in reducing and nonreducing conditions (NRC), and SDS-PAGE. Compared with WT, cells transfected with mutant AT and cells cotransfected with WT/mutant plasmids had an increase in intracellular high-molecular-weight complexes (HMWc) that can be observed in NRC.

Cotransfection of mutant and WT plasmids (1:1) was performed in HEK-EBNA cells to study a potential interaction between them. At 48 hours after transfection, we confirmed that the C-terminal variant AT had severely reduced secretion (mean relative secretion, 5.3% of WT; SD 4.0%); cotransfection experiments additionally revealed that the mutant plasmid interfered with the WT in a dominant-negative manner to severely impair AT secretion (mean relative secretion, 22.5% of WT; SD 19.1%) (Figure 3A). The analysis of the whole-cell lysates in reducing and nonreducing conditions revealed that the presence of the mutant protein, both in only-mutant and WT/mutant cotransfected cells, was associated with an increase in intracellular disulfide-linked, high–molecular weight complexes (Figure 3B).

### Biochemical and functional characterization of the recombinant p.Arg445Serfs*17 protein purified from the extracellular medium.

**Figure 4:**
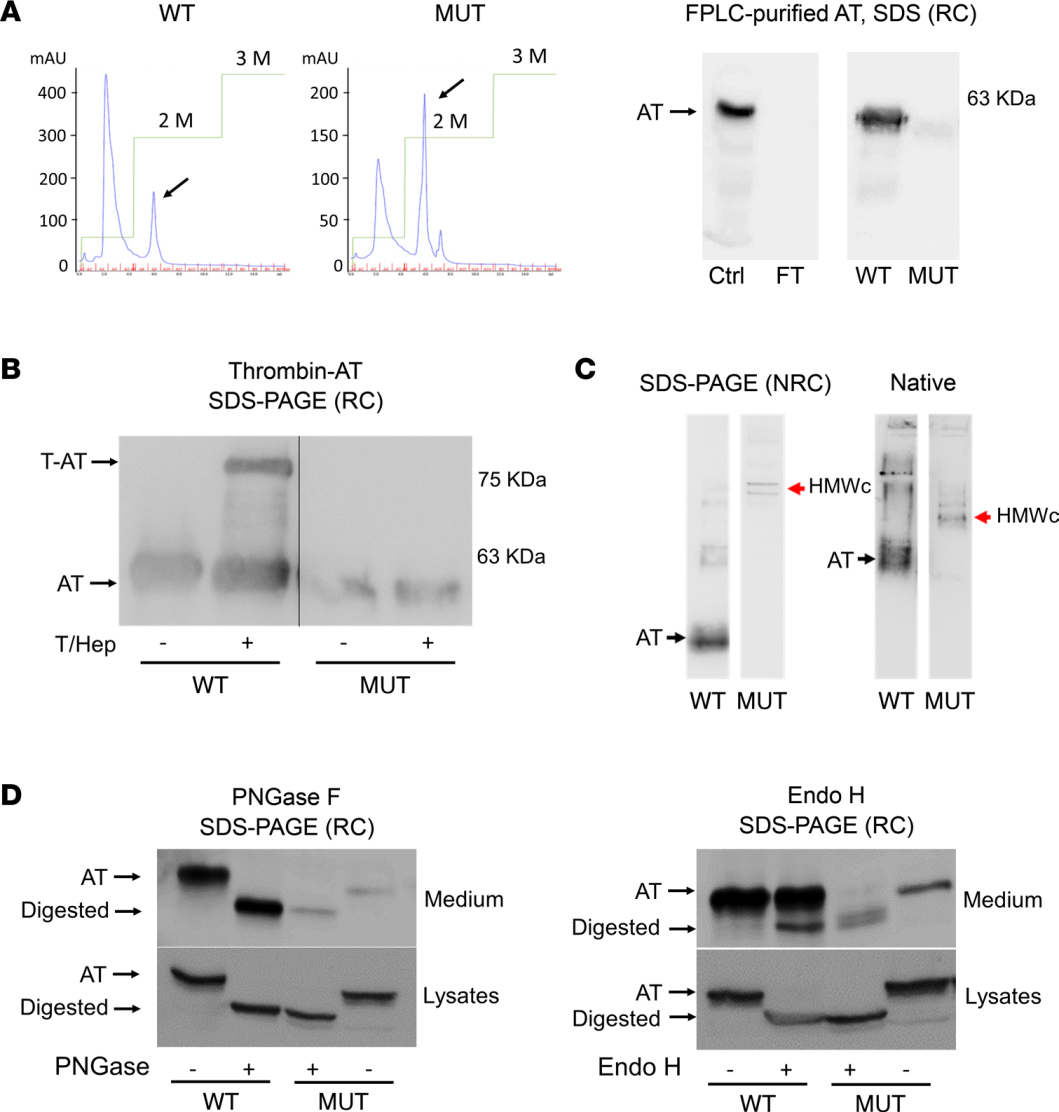
Characterization of the purified extracellular fraction of p.Arg445Serfs*17 in a recombinant model of transient expression in HEK-EBNA cells. (**A**) Purification of recombinant protein by FPLC. Elution profiles (blue curves) represent UV absorbance (*y* axis) according to 1 mL fractions (A1–A14, B1–B5; *x* axis). Saline concentration (green curves) is also shown. (**B**) Anti-FIIa activity assessment by identification of thrombin-AT (T-AT) induced complexes. The variant had no anticoagulant activity because no T-AT complexes were observed when incubated with (+) or without (-) thrombin and heparin (T/Hep) (*n* = 3 experiments). (**C**) Western blot for AT in purified medium in nonreducing conditions (NRC) SDS-PAGE and native gels. The variant formed disulfide-linked complexes (red arrows) (*n* = 3). (**D**) Qualitative N-glycosylation assessment with PNGase F and Endo H digestion of the purified extracellular fraction, and whole-cell lysates, of mutant (MUT) and WT protein. Intact AT and digested glycoproteins are pointed at (*n* = 2 experiments). Ctrl, healthy plasma control; FT, flow through; HMWc, high-molecular-weight complexes.

Because ectopic expression of p.Arg445Serfs*17 variant was compatible with the detection of a minimal fraction of mutant AT in the extracellular medium, we purified the recombinant protein from HEK-EBNA by heparin-affinity fast protein liquid chromatography (FPLC). The elution curve of p.Arg445Serfs*17 suggested that the variant preserved heparin affinity, because it was eluted at the same fraction than the WT AT, corresponding to forms with high heparin affinity (Figure 4A). However, variant AT did not retain inhibitory activity against thrombin (no thrombin–AT complexes were detected after incubation with thrombin and heparin) and had null anti-FXa activity assessed by chromogenic assay (Figure 4B). Analysis in different electrophoretic conditions revealed that, although the mobilities under denaturing, reducing conditions of WT and mutant AT were similar, the mutant protein formed disulfide-linked, high–molecular weight complexes under denaturing, nonreducing and native conditions (Figure 4C).

Enzymatic digestion with peptide-N-glycosylase F (PNGase F) and endo-β-*N*-acetylglucosaminidase H (Endo H) of both the WT and the mutant proteins also was performed to qualitatively assess their N-glycosylation state, a major post-translational modification of AT that takes place in the ER-Golgi system. With PNGase F, both the WT and the mutant AT showed the same (higher) electrophoretic migration pattern, identical at the extracellular and intracellular compartments, indicating that both ATs were equivalently N-glycosylated at the ER. Nevertheless, after using Endo H, although most of the secreted WT AT was not digested, nearly all the mutant protein detected in the extracellular medium was digested. This pattern was also observed in the whole-cell lysates of WT and mutant AT and suggested that, unlike the WT, whose N-glycans acquire late modifications at the Golgi, the high–molecular weight complexes of p.Arg445Serfs*17 that are minimally detected in the extracellular medium might not be secreted through the Golgi but be the result of the unspecific release of ER-retained polymers (Figure 4D).

### 1+ Frameshift AT variants may be secreted in an inactive form, but the consequences of the aberrant C-terminus depend on the frameshift location.

**Figure 5:**
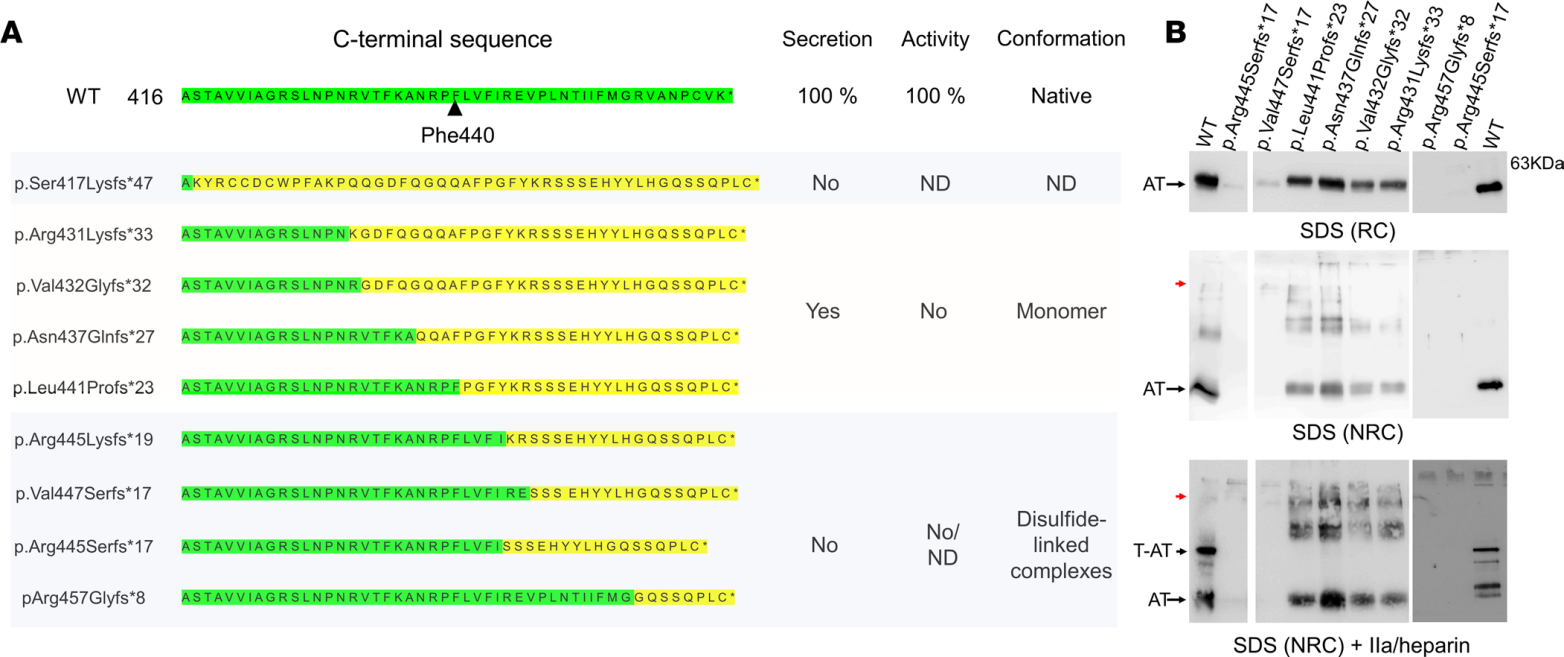
Analysis of 1+ frameshift C-terminal AT variants generated by site-directed mutagenesis in recombinant model of transient expression in HEK-EBNA cells. (**A**) Graphic summary. WT and aberrant sequences are highlighted in green and yellow, respectively. (**B**) Representative examples of recombinant variants detected in the conditioned medium in different electrophoretic conditions. 1+ Frameshift variants located upstream of Phe440 were efficiently secreted (≥50%) as monomers, but with no activity, whereas 1+ frameshift variants at Phe440 or after caused severe hyposecretion (null or minimal) and the formation of disulfide-bond complexes (red arrow) (*n* = 3 experiments). ND, not determined; NRC, nonreducing conditions; RC, reducing conditions; T-AT, thrombin-antithrombin complexes.

Overall, integrating results from our cohort with review of cases in the Human Gene Mutation Database, we identified a total of 21 INDELs in exon 7 of the *SERPINC1* (*n* = 13 deletions and 8 insertions), 18 of them causing frameshifts (Supplemental Tables 2 and 3). In addition to p.Arg445Serfs*17, 12 INDELs also lead to 1+ frameshift. All but 1 had no premature stop codons, so the frameshifts caused aberrant C-termini with the same characteristics of p.Arg445Serfs*17 but different lengths of the aberrant C-terminal sequence, ranging from 8 to 47 aa. On the basis of this observation, we generated 8 additional recombinant 1+ frameshift variants by site-directed mutagenesis (Figure 5). Then, recombinant plasmids were transiently expressed into HEK-EBNA cells.

We observed that the consequences of the aberrant C-termini depended on the frameshift site. If the frameshift site was downstream of WT Phe440, the variants were not secreted, similar to p.Arg445Serfs*17. Conversely, if the frameshift occurred at Phe440 or upstream, the variants were secreted as monomers, although less effectively than WT AT. However, these variants did not retain anticoagulant activity. An exception to this secretory profile was p.Ser417Lysfs*47, the variant with the most extended aberrant C-terminus, which was not secreted either (Figure 5).

### Pro441 is conserved in the aberrant C-termini of secreted frameshift variants, and Leu441Pro substitution in p.Arg445Serfs*17 and p.Arg445Lysfs*19 rescues variant secretion.

**Figure 6:**
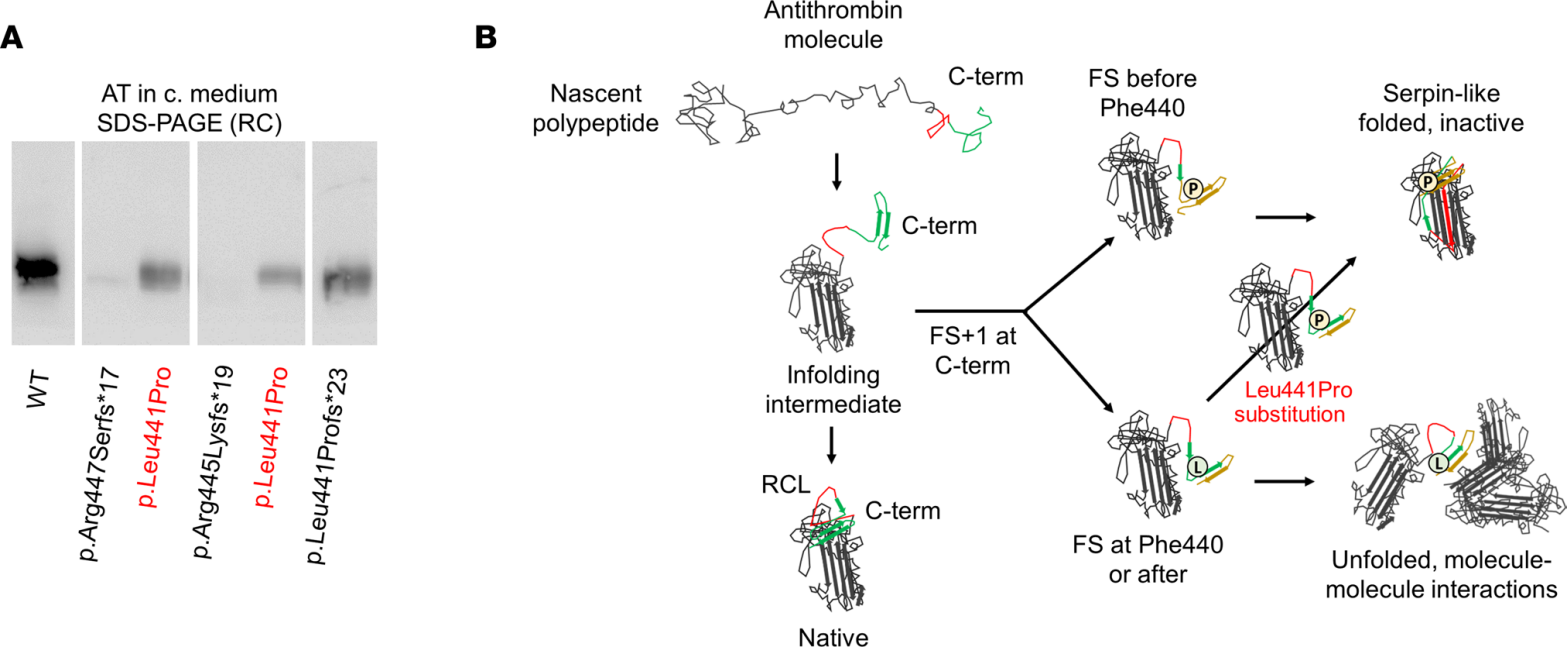
An aberrant proline (Pro441) shared by secreted variants rescued protein secretion when introduced in p.Arg445Serfs*17 and p.Arg445Lys*19 in a recombinant model of transient expression in HEK-EBNA cells. (**A**) Western blot for AT in reducing conditions (RCs), SDS-PAGE of the conditioned medium (c. medium) of cells transfected with 2 nonsecreted variants, p.Arg445Serfs*17 and p.Arg445Lysfs*19, and with the same plasmids after the introduction of Leu441Pro substitution by site-directed mutagenesis. Transient expression of these modified variants revealed partial rescue of protein secretion, reaching the levels of secreted C-terminal (C-term) variants such as p.Leu441Profs*23 (*n* = 3 experiments). (**B**) Graphic representation of the hypothesis about the effect of the aa changes. FS: frameshift; NRC, nonreducing conditions.

Comparative analysis of the primary sequences and the secretory patterns of the frameshift variants carrying different-extension, aberrant C-termini enabled us to identify a p.Pro441-Tyr444 sequence, shared by the aberrant C-termini of secreted variants, as a potential key sequence to allow protein secretion. We particularly focused on the Pro441 residue, considering the relevant impact of Pro losses and gains on AT folding and secretion (10). Therefore, we introduced the p.Leu441Pro substitution into 2 nonsecreted variants causing type I deficiency, p.Arg445Serfs*17 and p.Arg445Lysfs*19. This modification resulted in partial rescue of protein secretion of both p.Arg445Serfs*17 and p.Arg445Lysfs*19, facilitating their production as inactive monomers (Figure 6A).

## Discussion

Our results suggest a key structural relevance for the C-terminus of AT and potentially all serpins. In our work, by reviewing 1 of the largest and most genetically diverse centralized cohorts comprising 444 unrelated cases with AT deficiency, we identified 17 patients carrying 12 different *SERPINC1* variants involving the AT C-terminus (p.Val432-Lys464), all in exon 7 and not affecting the RCL. Biological characterization of C-terminal AT variants suggests that their phenotypic diversity can be divided into 2 distinct groups: (a) mutations located at s1C (p.Phe434-Pro439), which are missense and cause type II PE deficiency; and (b) mutations located at s4B-s5B (p.Phe440-Lys461) that are mainly INDELs and cause severe type I deficiency.

Previous reports support this dimorphism. Lane et al. (34) reported 6 different missense mutations affecting s1C causing type II PE deficiency. Soon thereafter, Emmerich et al. (27) reported 4 small INDELs in exon 7 causing type I deficiency that were grouped in a cluster at s4B (p.Phe440-Arg445). Moreover, they proposed the existence of an INDEL hotspot facilitated by repetitive DNA sequences affecting s4B. By integrating data from 12 different C-terminal variants (4 of them not previously reported, to our knowledge), our present work supports this hypothesis and shows that not only s4B but the whole s4B–s5B region constitutes an INDEL hotspot. Indeed, we have recognized the same repetitive DNA sequences suggested by Emmerich et al. (27) in the 3′UTR of *SERPINC1*, extending this hotspot to the whole p.Phe440-Lys461 polypeptide (Supplemental Figure 3).

Current knowledge of the serpin folding pathway, mainly based on experiments on AAT (35) and ovalbumin (36, 37), supports the relevance of the C-terminus of AT as well as the dual behavior observed among C-terminal variants. The proper insertion of the C-terminus is needed for achieving the native conformation (19). Consequently, a gene variant affecting the C-terminus, but permissive for completion of β-sheets B and C, may be compatible with secretion and native state, although transformation into a hyperstable state might be facilitated (19, 38). This may be the case for missense variants located at s1C that lead to type II PE deficiency and increased latent transition. On the other hand, a variant blocking the correct positioning of the C-terminus, and impairing its insertion into the serpin backbone, may directly have negative effects on folding and secretion (19, 39). Certainly, this might be the scenario of INDELs and some missense variants located at s4B–s5B that cause type I deficiency.

Certain variants interfering with the insertion of the C-terminus might additionally lead to the intracellular accumulation of serpin-like misfolded intermediates within the ER (40, 41), which are prone to interaction and polymerization (42). Serpinopathies are a subset of diseases associated with mutant serpin polymerization, ER retention, and eventual cell damage and organ degeneration. Familial encephalopathy with neuroserpin inclusion bodies and Z-AAT–driven liver cirrhosis are paradigmatic examples (42–44). A similar phenomenon underlies the nontoxic hepatic retention and plasma deficiency of several serpins, among which AT is included (29, 30, 45). We and other groups have already reported that this pathogenic mechanism occurs in the AT secretory machinery, causing protein aggregation and ER stress pathways (46–48). Here, we report, to our knowledge for the first time, how a variant in *SERPINC1*, p.Arg445Serfs*17, causes severe type I AT deficiency through a pathogenic mechanism of negative dominance involving intracellular blockade and ER dilation and fragmentation. The identification of this high-risk molecular defect could be of prognostic relevance and could assist antithrombotic management in this subset of patients. More studies should be conducted to explore this pathogenic mechanism in variants affecting other regions of AT rather than the C-terminus, including missense mutations causing type I deficiency, small in-frame INDELs leading to near-full-length variants, or, simply, cases with type I deficiency and very low AT activities in plasma (less than expected for heterozygous carriers).

Regarding the exact mechanism by which p.Arg445Serfs*17 impairs WT secretion, many observations strongly suggest that, as Z-AAT and C1-inhibitor variants (29, 30), hetero-polymerization of mutant and WT proteins is the most likely cause. Direct clinical data from our patients suggest that restricted-to-variant protein aggregation and ER retention do not totally explain the dominant-negative effect on WT AT, because patients did not present with hypertransaminasaemia or analytical abnormalities in other plasmatic proteins produced by the liver, including the serpin AAT (data not shown). Consequently, variant AT might be impairing intracellular trafficking of WT AT in a specific way. On the basis of our observations in a recombinant model, we propose that mutant AT might be directly sequestering the WT protein into ER aggregates. Classic mechanisms suggested for serpin polymerization (49) and/or the formation of high–molecular weight complexes through disulfide bonds between mismatched Cys (as we observed when purifying the aberrant protein) could help explain the observed dominant-negative effect. In fact, Emmerich et al. (50) reported 3 AT variants that presented a small fraction of disulfide-linked, high–molecular weight complexes in plasma. We identified these complexes in 1 C-terminal missense variant from our cohort (p.Gly456Arg) (32). However, we detected the same complexes not in plasma samples from patients carrying p.Arg445Serfs*17 but only in the highly efficient recombinant model, so it seems that the majority of the complexes are intracellularly retained and degraded. Further characterization of this infective phenomenon and the exploration of the potential benefit of chemical or pharmacological chaperones will provide a better understanding of these pathways and of the possibility of targeted therapies.

We have expanded our study to previously described 1+ frameshift, C-terminal AT variants (26, 27, 51–53) other than p.Arg445Serfs*17. The analysis of these variants revealed that all were of similar size to the WT molecule but a different extension of the aberrant C-terminus. If the frameshift takes place at Phe440 or upstream, mutations affecting p.Val432-Phe440, despite having a long, aberrant C-terminus, will be efficiently secreted, although without anticoagulant activity, probably because they adopt serpin-like folding, but far from the metastable conformation, with an exposed RCL that is required for the mousetrapping inhibitory mechanism. This is likely explained by the presence of a proline and a cysteine (Pro459 and Cys461), rather than Pro461 and Cys462 in the WT homologous, with the last residue involved in the C-terminal disulfide bond (7, 18, 20). However, and intriguingly, if the 1+ frameshift occurs downstream of Phe440, as in p.Arg445Serfs*17, despite having a shorter aberrant C-terminus, it might cause severely impaired secretion, no anticoagulant activity, and even a dominant-negative effect on WT AT. That p.Leu441Pro substitution partially rescued p.Arg445Serfs*17 and p.Arg445Lysfs*19 variant secretion suggests that Pro441 provokes a torsion of the aberrant C-terminus that minimizes hydrophobic core exposition, prevents intracellular polymerization, and allows the reasonable serpin-like folding compatible with secretion (7, 16–18, 34) (Figure 6B). These preliminary finding encourage research on the use of small molecules to mimic this conformational change (54) as a potential way to alleviate the severity of these cases.

Our cellular model consisted of the transient expression of recombinant cDNA within a constitutive expression cassette in HEK-EBNA and CHO lines. We intentionally avoided the use of hepatic cells in order to prevent interference by endogenously expressed AT (6). Other approaches, such as the use of more stable means of transgene expression (55), the use of transfected or CRISPR/Cas9 gene–edited, AT-expressing hepatic lines, or the use of hepatocyte-like cells from reprogrammed, human, induced pluripotent stem cells carrying the specific *SERPINC1* defect from patients (56–58), could be explored in future works for a better understanding of the underlying pathogenic mechanisms.

In conclusion, we have shown that the C-terminus plays a key structural role in AT and potentially in all serpins. Variants at the C-terminus of AT generate a considerable biological and clinical heterogeneity strongly related with the localization of the variant. 1+ Frameshift at the C-terminus of AT might be compatible with protein secretion but not function. Nevertheless, the consequences of 1+ frameshift dramatically veer if it takes place downstream Phe440, because it provokes protein aggregation, ER retention, and even a dominant-negative effect on WT protein. Finally, p.Arg445Serfs*17 constitutes, to our knowledge, the first description of a mutation in *SERPINC1* causing severe AT deficiency through a dominant-negative effect that could be of prognostic significance. Researchers should explore this pathogenic mechanism in variants affecting other regions of AT and investigate the use of gene editing and/or pharmacological chaperones as a new therapeutic tool in this subset of mutations.

## Methods

### Study cohort.

The study was conducted in a cohort of 444 unrelated participants with congenital AT deficiency who were enrolled during a 23-year period (1998–2021). Briefly, patients with potential AT deficiency diagnosed by functional methods (AT activity <80%) were referred to our center from more than 20 Spanish and other European hospitals for characterization. Recommendations to avoid artefactual and acquired deficiency were followed (12, 59). Demographic and clinical data of thrombotic events and possible risk factors were extracted from medical records. Venous thrombotic events were objectively diagnosed and confirmed through routine imaging procedures.

Blood was collected from the antecubital vein into citrate tubes (109 mmol/L) at local hospitals and processed within 24 hours after extraction. A new sample was obtained from each referred patient, and this was immediately delivered to our center and processed within 48 hours after extraction to validate AT deficiency.

### Measurement of plasma AT activity and levels.

AT activity against FXa and thrombin (FIIa) was determined by chromogenic methods in citrated plasma. Anti-FXa assays were performed with heparin, bovine FXa, and S-2765 chromogenic substrate (HaemosIL TH, Instrumentation Laboratory). Anti-FIIa assays were performed with unfractionated heparin, bovine thrombin, and S-2238 chromogenic substrate (Werfen) (31). AT antigen levels were measured by immunodiffusion (Laurell Technologies) (31). Values are expressed as the percentage of normal pool plasma from 100 healthy control participants.

### Protein electrophoresis and Western blot.

Western blot for AT was performed as described previously (32). Briefly, PAGE was conducted under denaturing (reducing or nonreducing) and native (with or without 6 M urea) conditions. After separation, proteins were transblotted onto a PVDF membranes. AT was immunostained with rabbit anti–human AT polyclonal Ab (no. A9522, Sigma-Aldrich), and then donkey anti–rabbit IgG conjugated with HRP (GE Healthcare), and detected with an ECL kit (Amersham Biosciences). The amount of secreted protein to the conditioned medium detected in denaturing, reducing conditions was quantified by densitometry, using ImageJ software (NIH), and expressed as a relative secretion (as a percentage) compared with WT AT cells (reference category).

### N-glycosylation assessment.

N-glycosylation was evaluated by comparative Western blots in basal conditions and after digestion with Endo H (New England Biolabs), following manufacturer’s instructions, and with PNGase F (Sigma-Aldrich). Briefly, for the second enzymatic digestion, cell supernatants (≤200 μg of glycoprotein in a volume of 35 μL) were denatured with 10 μL of 250 mM phosphate buffer and 2.5 μL of 2% SDS with 1 M 2-mercaptoethanol, heated at 100°C for 5 minutes and cooled. TRITON X-100 (2.5 μL of 15%) was added. Then, 2.0 μL of PNGase F (≥5,000 units/mL) was added and incubated overnight at 37°C. Samples were run in SDS-PAGE and detected as described above.

### Genetic analysis.

Genomic DNA was purified by standard procedures. Gene variants in *SERPINC1* were determined by sequencing the 7 exons and flanking regions, as well as the promoter, using primers and conditions described previously (60). Structural variants in *SERPINC1* were evaluated by multiplex ligation-dependent probe amplification (MLPA) analysis using the SALSA MLPA Kit P227 *SERPINC1* (MRC_Holland).

### Recombinant plasmids.

The pCEP4-S137A plasmid, generously provided by J. Huntington (Cambridge Institute for Medical Research, University of Cambridge, Cambridge, United Kingdom), contains the cDNA of human *SERPINC1* but in the p.Ser137Ala AT background, which only produces the β-glycoform of AT, as a way to reduce glycosylation heterogeneity and to facilitate purification. Variant AT plasmids were generated by site-directed mutagenesis (61), using the Stratagene Quick Change Site-Directed Mutagenesis kit (Agilent Technologies) using the appropriate primers.

Expression constructs encoding YFP-tagged mutant and WT AT were generated by replacing the coding sequence of AAT in YFP-AAT (33) with the coding sequence of the signal peptide–cleaved form of human AT (aa 33–464 in the WT) using Gibson Assembly (New England BioLabs). The resultant YFP-AT constructs encoded the N-terminal cleavable signal of human calreticulin to facilitate ER localization, followed by the fluorescent protein YFP, followed by a (Gly4Ser)3 linker sequence, followed by AT and a stop codon. YFP-tagged M and Z forms of AAT and mCherry-ER constructs have been previously described (33).

### Cell lines, cultures, and protein purification.

HEK-EBNA and CHO cell lines were grown in DMEM (Invitrogen) supplemented with 10% FBS, glutamine (1 mmol/L), penicillin (120 μg/mL), streptomycin (100 μg/mL), and glucose (4.5 μg/mL) to 50% to 70% confluence at 37°C and 5% CO_2_ in a humidified incubator.

Cells were transfected with Lipofectamine LTX & PLUS reagent (Invitrogen), following the manufacturer’s recommendations. After 24 hours, the medium was changed for chemically defined CHO (Invitrogen), supplemented with glutamine (4 mmol/L) and geneticin (250 μg/mL). Depending on the purpose of the experiment, cells were grown for 2 to 10 days, but culture medium was changed every 2 days.

For analysis of recombinant protein secretion, activity, and conformation, medium was collected and cells were washed with sterile PBS and then lysed with 50 μL of lysis buffer (pH 7.5; Tris-HCl 20 mmol/L; NaCl 150 mmol/L; Na_2_EDTA 1 mmol/L; EGTA 1 mmol/L; NP-40 1%; sodium deoxycholate 1%; sodium pyrophosphate 2.5 mmol/L; B-glycerophosphate 1 mmol/L; Na_3_VO_4_ 1 mmol/L; and leupeptin 1 μg/mL). Purification of recombinant proteins secreted to the medium was performed by heparin affinity FPLC followed by ion-exchange chromatography, as described elsewhere (61). Saline elution was performed in 3 steps, with escalated ascending concentrations of NaCl (0.5 M, 2 M, and 3 M).

### Confocal laser microscopy and live-cell imaging.

CHO cells were transfected with mCherry-ER and YFP-AT or YFP-AAT constructs in 35 mm live-cell imaging dishes (MatTek) and, 24 hours later, imaged in tissue culture medium supplemented with 25 mM HEPES, pH 7.0 to 7.6. Live-cell imaging was performed as reported previously (33, 62). Confocal micrographs were acquired using a Zeiss LSM780 laser scanning confocal microscope (Zeiss) using a ×63 1.4NA oil immersion objective. YFP- and mCherry-tagged proteins were excited at 514 nm and 561 nm, respectively, and images were captured with 1,028 × 1,028 pixel frame size.

### Gold immunolabeling and transmission electron microscopy.

HEK-EBNA cells were transfected with WT or mutant AT plasmids and, 96 hours later, fixed with 2% paraformaldehyde and 0.2% glutaraldehyde in 0.1 M sodium phosphate buffer, pH 7. Sample were processed for cryo-ultramicrotomy to enable ultrastructural analysis, and colloidal gold immunolabeling of AT was performed. Samples were rinsed and blocked in blocking solution (PBS-BSA 1%) for 5 minutes and incubated with rabbit anti–human AT polyclonal Ab (1:250, Dako Diagnostics) for 30 minutes. Then, sections were washed in 0.1% Tween-TBS and incubated with 10 nm of protein A–conjugated colloidal gold in Tween-TBS (1:40) (Sigma-Aldrich). After labelling, the sections were treated with 1% glutaraldehyde, counterstained with uranyl acetate at pH 7, and embedded in methyl cellulose-uranyl acetate at pH 4 (9:1). Grids were examined with a Philips Tecnai 12 electron microscope.

### In silico modeling and molecular dynamics.

Predictions of changes in protein structure were performed with Swiss-Model (https://swissmodel.expasy.org/) and AlphaFold (https://alphafold.ebi.ac.uk/) (63), and models were visualized with UCSF Chimera (https://www.cgl.ucsf.edu/chimera/). Crystal structure of AT was obtained for simulation from the Protein Data Bank (PDB) (PDB identification no. 2ANT). Changes in protein molecular weight and isoelectric point were also calculated with the isoelectric point calculation tool (http://isoelectric.ovh.org/).

Molecular dynamics simulations of selected variants and WT AT were performed as previously described (64, 65). Simulation setups are specified in Supplemental Table 4.

### Statistics.

Statistical analysis was performed using GraphPad Prism (GraphPad Software) and IBM SPSS Statistics 21 (IBM Corp.). Descriptive analysis of qualitative and quantitative variables included percentages and mean ± SD, respectively. Pearson’s χ^2^ test and Fisher’s exact test were used for comparison of proportions. The Shapiro-Wilk test was used for testing normality of continuous variables. The parametric Student’s *t* test and nonparametric Mann-Whitney *U* test were used for comparison of 2 means. Differences with a 2-tailed *P* values less than 0.05 were considered statistically significant.

### Study approval.

This study was approved by the local ethics committee of Morales Meseguer University Hospital, and performed in accordance with the 1964 Declaration of Helsinki and its later amendments. All included participants gave their written informed consent to enter the study.

## Author contributions

CBP, MT, MEMB, BMB, AM, JP, and RCR performed protein and genetic assays and recombinant model experiments and analyzed data. JAMM performed electron microscopy. MT, JEC, and SJM generated YFP-tagged serpins and performed confocal microscopy. CBP, PGR, and MEMB conducted in silico modelling. HPS performed molecular dynamics simulations. CBP created figures and wrote the manuscript. VV, MLL, MEMB, and JC designed and supervised the work, discussed the results, and wrote the manuscript. All authors revised the manuscript.

## Supplementary Material

Supplemental data

## Figures and Tables

**Table 1:**
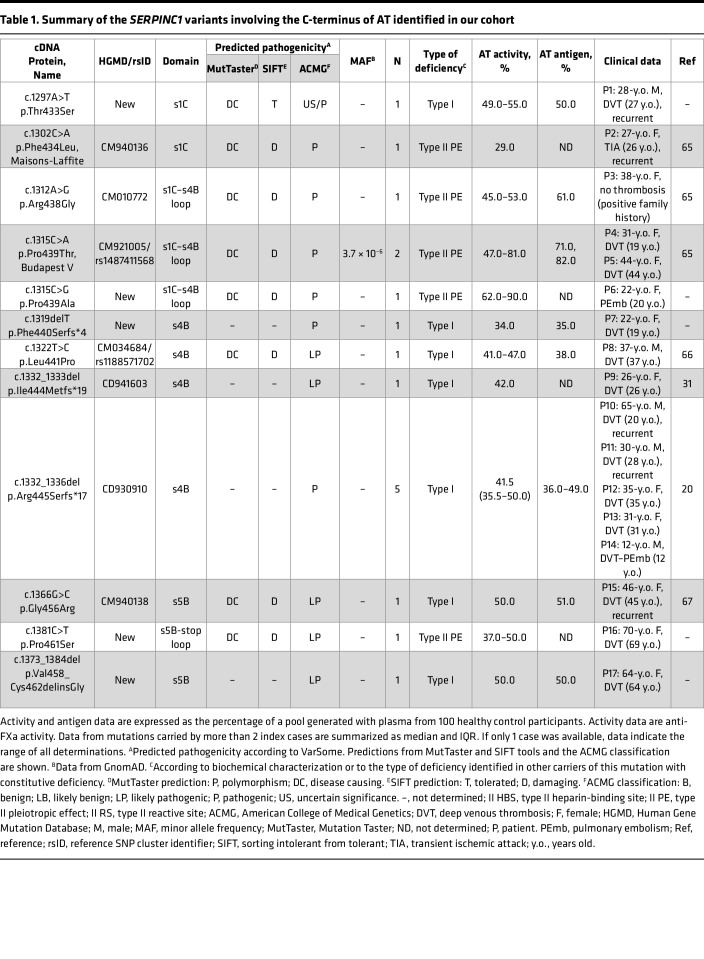
Summary of the *SERPINC1* variants involving the C-terminus of AT identified in our cohort

## Version 1 10/10/2022

Electronic publication
