# Supplementary material for: Full-length antithrombin frameshift variant with aberrant C-terminus causes endoplasmic reticulum retention with a dominant-negative effect
Source: JCI Insight. 2022 Oct 10;7(19):e161430. doi: 10.1172/jci.insight.161430 (PMC9675572; doi:10.1172/jci.insight.161430)
Supplement: Supplemental data [file jciinsight-7-161430-s136.pdf]

SUPPLEMENTARY MATERIAL

Table of contents

Supplementary Tables

Supplementary Table 1..2

Supplementary Table 2..3

Supplementary Table 3..5

Supplementary Table 4..7

Supplementary Figures

Supplementary Figure 1...8

Supplementary Figure 2...9

Supplementary Figure 3...10

Supplementary Table 1. Common *SERPINC1* haplotype in unrelated carriers of c.1332-1336delAAGAG (n=3)

dbsnp	MAF
rs2227612	0.135
rs677	0.110
rs148783362	0.013
rs61827936	0.133
rs1799876	0.470
rs5878 synonymous	0.464
rs5877 synonymous	0.450
rs2227597	0.237
rs2227596	0.349
rs2227595	0.126
rs2227594	0.237
rs2227593	0.238
rs2227590	0.243

MAF: Minor allele frequency.

Supplementary Table 2. Summary of *SERPINC1* exon 7 deletions

ID	cDNA<br>-(deleted nucleotides)	Protein<br>(reading frame)†	Amino acid sequence encoded by cDNA (exon 7)‡
1	c.1237-1239del<br>-GAA	p.Glu413del<br>(*inframe*)	407 VNEEG S A A A S T A V V I A G R S L N P N R V T F K A N R P F L V F I R E V P L N T I I F M G R V A N P C V K *
2	c.1264del<br>-A	p.Ile422Leufs*5<br>(+2)	407 VNEEG S E A A A S T A V V L L A V R * T P T G * L S R P T G L S W F L * E K F L * T L L S S W A E * P T L V L S K
3	c.1272-1274del<br>-CCG	p.Arg425del<br>(*inframe*)	407 VNEEG S E A A A S T A V V I A G S L N P N R V T F K A N R P F L V F I R E V P L N T I I F M G R V A N P C V K *
4	c.1312del<br>-A	p.Arg438Glyfs*6<br>(+2)	407 VNEEG S E A A A S T A V V I A G R S L N P N R V T F K A N G L S W F L * E K F L * T L L S S W A E * P T L V L S K
5	c.1319del<br>-T	p.Phe440Serfs*4<br>(+2)	407 VNEEG S E A A A S T A V V I A G R S L N P N R V T F K A N R P S W F L * E K F L * T L L S S W A E * P T L V L S K
6	c.1326del<br>-T	p.Phe443Leufs*1<br>(+2)	407 VNEEG S E A A A S T A V V I A G R S L N P N R V T F K A N R P F L V L * E K F L * T L L S S W A E * P T L V L S K
7	c.1332_1333del<br>-AA	p.Ile444Mfs*19<br>(+1)	407 VNEEG S E A A A S T A V V I A G R S L N P N R V T F K A N R P F L V F M R S S S E H Y Y L H G Q S S Q P L C *
8	c.1332_1336del<br>-AAGAG	p.Arg445Serfs*17<br>(+1)	407 VNEEG S E A A A S T A V V I A G R S L N P N R V T F K A N R P F L V F I S S S E H Y Y L H G Q S S Q P L C *
9	c.1347del<br>-G	p.Asn450Thrfs*8<br>(+2)	407 VNEEG S E A A A S T A V V I A G R S L N P N R V T F K A N R P F L V F I R E V P L T L L S S W A E * P T L V L S K
10	c.1366del<br>-G	p.Gly456Alafs*2<br>(+2)	407 VNEEG S E A A A S T A V V I A G R S L N P N R V T F K A N R P F L V F I R E V P L N T I I F M A E * P T L V L S K
11	c.1373_1384del<br>-TAGCCAACCCTT	p.Val458Glyfs*3<br>(+2)	407 VNEEG S E A A A S T A V V I A G R S L N P N R V T F K A N R P F L V F I R E V P L N T I I F M G R G V K *
12	c.1375-1383del<br>-GCCAACCCT	p.Ala460-Pro462del<br>(inframe)	407 VNEEG S E A A A S T A V V I A G R S L N P N R V T F K A N R P F L V F I R E V P L N T I I F M G R V C V K *
13	c.1390-1393del<br>-AAGT	p.Stop465Metfs*13<br>(+2)	407 VNEEG S E A A A S T A V V I A G R S L N P N R V T F K A N R P F L V F I R E V P L N T I I F M G R V A N P C V K M F L F F A P L P I F G L *

† The effect of the INDEL on the reading frame is indicated (*inframe*, frameshift +1 or frameshift +2).
‡ Open reading frame is highlighted in bold, whereas the non-codifying sequence is highlighted in italics. The aa sequence in which the reading frame changes is highlighted in red.
cDNA: complementary DNA. *: Stop codon.

Supplementary Table 2. Summary of *SERPINC1* exon 7 deletions (cont.)

ID	MW (KDa)†	IP†	N (overall)‡	N (our cohort)	Age of 1st thromb. event (y.o.)§	AT activity (%)¶	HGMD®	Ref.
1	52.47	6.290	1	0	37	48	CD994760	Tsuda (1999)
2	48.3	5.422	1	0	.	.	CD982444	Jochmans (1998)
3	52.45	5.890	2	1	30	55	CD036071	Raja (2003)
4	50.19	5.625	1	0	.	.	CD117608	Celinska (2011)
5	50.28	5.749	1	1	19	34	New	.
6	50.22	5.749	1	0	.	52	CD930909	Chowdhury (1993)
7	52.44	5.860	2	1	.	50	CD941603	Emmerich (1994)
8	52.27	5.737	6	5	28 (16-45)	39 (33-51)	CD930910	Millar (1993)
9	51.85	5.638	1	0	.	.	CD061469	Schleithoff (2006)
10	51.88	5.638	1	0	.	.	CD024464	Fu (2002)
11	52.17	6.080	1	1	.	75	New	.
12	52.32	6.074	1	0	.	.	CD961791	Perry (1996)
13	108.72	6.070	1	0	.	.	CD125926	Castaldo (2012)

† Molecular weight (MW) and isoelectric point (IP) estimated by *Protein isoelectric point calculator* software (http://isoelectric.ovh.org/).
‡ Number (N) of all cases, including the first reported in literature.
§ Age of first thrombotic event. If more than one case, median and interquartile range are shown.
¶ Antithrombin activity. If more than one case, median and interquartile range are shown.
AT: antithrombin. HGMD®: Human Genetic Mutation Database. MW: molecular weight. N: number of cases. Ref: reference.

Supplementary Table 3. Summary of *SERPINC1* exon 7 insertions

ID	cDNA	Protein (reading frame)†	Amino acid sequence encoded by cDNA (exon 7)‡
14	c.1233dupC	p.Ser412Glnfs*1 (+1)	407 VNEEG Q * _S S C K Y R C C D C W P F A K P Q Q G D F Q G Q Q A F P G F Y K R S S S E H Y Y L H G Q S S Q P L C_ *
15	c.1247dupC	p.Ser417Lysfs*47 (+1)	407 VNEEG S E A A A K Y R C C D C W P F A K P Q Q G D F Q G Q Q A F P G F Y K R S S S E H Y Y L H G Q S S Q P L C *
16	c.1255-56ins ACCG	p.Ala419Aspfs*46 (+1)	407 VNEEG S E A A A S T D R C C D C W P F A K P Q Q G D F Q G Q Q A F P G F Y K R S S S E H Y Y L H G Q S S Q P L C *
17	c.1292dupG	p.Val432Glyfs*32 (+1)	407 VNEEG S E A A A S T A V V I A G R S L N P N R G D F Q G Q Q A F P G F Y K R S S S E H Y Y L H G Q S S Q P L C *
18	c.1320-21insA	p.Phe440Leufs*24 (+1)	407 VNEEG S E A A A S T A V V I A G R S L N P N R V T F K A N R P L P G F Y K R S S S E H Y Y L H G Q S S Q P L C *
19	c.1357dupA	p.Ile453Asnfs*11 (+1)	407 VNEEG S E A A A S T A V V I A G R S L N P N R V T F K A N R P F L V F I R E V P L N T I N L H G Q S S Q P L C *
20	c.1366dupG	p.Arg457Glyfs*8 (+1)	407 VNEEG S E A A A S T A V V I A G R S L N P N R V T F K A N R P F L V F I R E V P L N T I I F M G Q S S Q P L C *
21	c.1390dupA	p.Stop465Valfs*18 (+2)	407 VNEEG S E A A A S T A V V I A G R S L N P N R V T F K A N R P F L V F I R E V P L N T I I F M G R V A N P C V K V K C S Y S L H L F L F L V C E Q K *

† The effect of the INDEL on the reading frame is indicated (*inframe*, frameshift +1 or frameshift +2).
‡ Open reading frame is highlighted in bold, whereas the non-coding sequence is highlighted in italics. The aa sequence in which the reading frame changes is highlighted in red.
cDNA: complementary DNA. *: Stop codon.
.

Supplementary Table 3. Summary of *SERPINC1* exon 7 insertions (cont.)

ID	MW (KDa)†	IP†	N (overall)‡	N (our cohort)	Age of 1st thromb. event (y.o.)§	AT activity (%)¶	HGMD®	Ref.
14	46.99	5.414	1	1	50	56	.	.
15	52.78	5.630	1	0	30	47	CI041962	David (2004)
16	52.79	5.453	1	0	45	50	CI941833	Emmerich (1994)
17	52.46	5.633	1	0	23	37	CI041963	David (2004)
18	52.51	6.001	1	0	30	.	CI941834	Gandrille (1991)
19	52.45	5.807	1	0	.	69	CI941835	Chowdhury (1993)
20	52.48	5.752	1	0	9	52	CI941836	Olds (1991)
21	57.73	5.138	1	0	.	50	CI011219	Lane (1997)

† Molecular weight (MW) and isoelectric point (IP) estimated by *Protein isoelectric point calculator* software (http://isoelectric.ovh.org/).
‡ Number (N) of all cases, including the first reported in literature.
§ Age of first thrombotic event. If more than one case, median and interquartile range are shown.
¶ Antithrombin activity. If more than one case, median and interquartile range are shown.
AT: antithrombin. HGMD®: Human Genetic Mutation Database. MW: molecular weight. N: number of cases. Ref: reference.

Supplementary Table 4. Molecular dynamics setup conditions

Condition	Initial setup
Force field	*Amber99sb*
Number of sol/ atoms/ ions/molecules	*54447/ 61318 /4/ 1*
Cut-off	*0.9*
Water model	*tip3p*
Temperature	*300*
Non-bonded int	*PME*
Ensemble	*NPT2*
Simulation time	*100 – 680 ns*
Integration step	*0.002*
Box type	*dodecahedron*
P coupling	*Parrinello-Rahman*
T coupling	*V-rescale*

Supplementary Figures

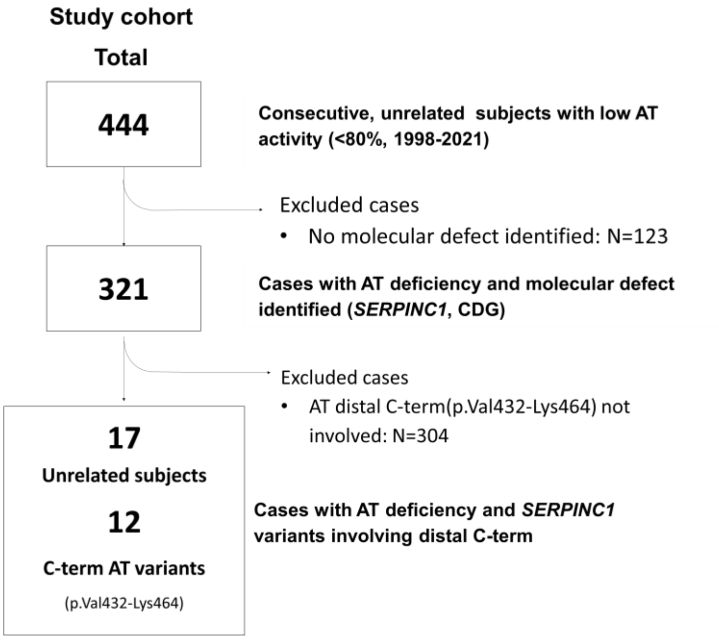

Supplementary Figure 1. Study design, patient flow chart and summary of SERPINC1 molecular defects identified. *AT: antithrombin. C-term: C-terminal/C-terminus. CDG: congenital disorders of glycosylation.*

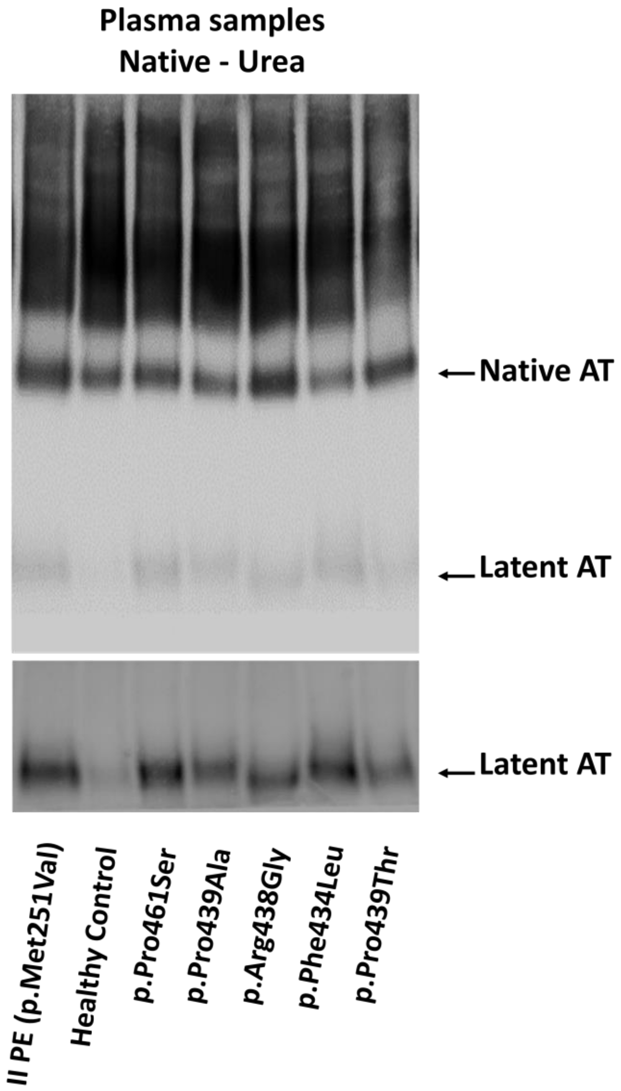

Supplementary Figure 2. Native-urea gels of C-terminal variants associated to type II PE antithrombin deficiency due to an increase in latent AT. Western blot for AT in plasma samples of carriers of p.Phe434Leu, p.Arg438Gly, p.Pro439Thr, p.Pro439Thr, and p.Pro461Ser variants after electrophoresis in native conditions with 6 M urea. Samples from a healthy subject and a positive II PE control, corresponding to a type II PE variant that causes increased latent transformation, but is not located in *SERPINC1* exon 7 (p.Met251Val), are also shown. Latent AT, that can be better appreciated in the lower panel, with higher exposition, is pointed with arrows. *AT: antithrombin. II PE: II pleiotropic deficiency.*

```
                 +C   -GAA      +C     +ACCG       -A         -CGT
1219 GTAAATGAAGAAGGCAGTGAAGCAGCTGCAAGTACCGCTGTTGTGATTGCTGGCCGTTCGCTAAACCCC 1280
407  -V--N--E--E--G--S--E--A--A--A--S--T--A--V--V--I--A--G--R--S--L--N--P-   429


                                -T          -AA
           +G             -A      +A       -T -AAGAG          -G
1281 aacaGGGTGACTTTCAAGGCCaacaGGCCTttcctGGTTTTTATAAGAGAAGttcctCTGaacaCTATT 1356
  430  -N--R--V--T--F--K--A--N--R--P--F--L--V--F--I--R--E--V--P--L--N--T--I-
      452


            -G        -GCCAACCCT   +A
    +A      +G     -TAGCCAACCCTT   -AAGT
1357 ATCTTCATGGGCAGAGTAGCCAACCCTTGTGTTAAGTAAAATGTTCTTATTCTTTGCACCTCttcctTA 1417
453  -I--F--M--G--R--V--A--N--P--C--V--K--*-………………………………………………………………………


1418 TTTTTGGTTTGTGaacaGAAGTAAAAATAAATACAAACTACTTCCATCTCA……………………………………… 1468
     …………………………………………………………………………………………………………………………………………………………
```

Supplementary Figure 3. Cluster of INDEL in exon 7 of *SERPINC1*. Nucleotide and peptide sequences showing the 21 deletions/insertions collected from HGMD® and from our cohort. Deleted and duplicated nucleotides are preceded by the signs "–" and "+", respectively. The repetitive sequences identified by Emmerich et al. between p.Phe440-Arg445, which we have also identified in the 3'UTR region, are shown in small letter.

HGMD®: *Human Gene Mutation Database.* 3'UTR: 3' *Untraslated región.*
